# Posttranslationally modified progesterone receptors direct ligand-specific expression of breast cancer stem cell-associated gene programs

**DOI:** 10.1186/s13045-017-0462-7

**Published:** 2017-04-17

**Authors:** Todd P. Knutson, Thu H. Truong, Shihong Ma, Nicholas J. Brady, Megan E. Sullivan, Ganesh Raj, Kathryn L. Schwertfeger, Carol A. Lange

**Affiliations:** 1 Departments of Medicine (Division of Hematology, Oncology, and Transplantation) and Pharmacology, Masonic Cancer Center, University of Minnesota, Delivery Code 2812, Cancer and Cardiovascular Research Building, 2231 6th St SE, Minneapolis, MN 55455 USA; 2 Department of Urology, UT Southwestern Medical Center at Dallas, 5323 Harry Hines Blvd, J8.130C, Dallas, TX 75390-9110 USA; 3 Microbiology, Immunology, and Cancer Biology Graduate Program, University of Minnesota, Minneapolis, MN 55455 USA; 4 Department of Pathology, Evanston Hospital, University of Chicago, NorthShore University HealthSystem, Evanston, IL 60201 USA; 5 Department of Laboratory Medicine and Pathology, Masonic Cancer Center, University of Minnesota, Minneapolis, MN 55455 USA

**Keywords:** Progesterone receptor (PR), Phosphorylation, ERK/MAP kinase (MAPK), SUMOylation, Antiprogestin, Onapristone, Estrogen receptor (ER), Breast cancer, RUNX2, Cancer stem cells

## Abstract

**Background:**

Estrogen and progesterone are potent breast mitogens. In addition to steroid hormones, multiple signaling pathways input to estrogen receptor (ER) and progesterone receptor (PR) actions via posttranslational events. Protein kinases commonly activated in breast cancers phosphorylate steroid hormone receptors (SRs) and profoundly impact their activities.

**Methods:**

To better understand the role of modified PRs in breast cancer, we measured total and phospho-Ser294 PRs in 209 human breast tumors represented on 2754 individual tissue spots within a tissue microarray and assayed the regulation of this site in human tumor explants cultured ex vivo. To complement this analysis, we assayed PR target gene regulation in T47D luminal breast cancer models following treatment with progestin (promegestone; R5020) and antiprogestins (mifepristone, onapristone, or aglepristone) in conditions under which the receptor is regulated by Lys388 SUMOylation (K388 intact) or is SUMO-deficient (via K388R mutation to mimic persistent Ser294 phosphorylation). Selected phospho-PR-driven target genes were validated by qRT-PCR and following RUNX2 shRNA knockdown in breast cancer cell lines. Primary and secondary mammosphere assays were performed to implicate phospho-Ser294 PRs, epidermal growth factor signaling, and RUNX2 in breast cancer stem cell biology.

**Results:**

Phospho-Ser294 PR species were abundant in a majority (54%) of luminal breast tumors, and PR promoter selectivity was exquisitely sensitive to posttranslational modifications. Phospho-PR expression and target gene programs were significantly associated with invasive lobular carcinoma (ILC). Consistent with our finding that activated phospho-PRs undergo rapid ligand-dependent turnover, unique phospho-PR gene signatures were most prevalent in breast tumors clinically designated as PR-low to PR-null (luminal B) and included gene sets associated with cancer stem cell biology (*HER2*, *PAX2*, *AHR*, *AR*, *RUNX*). Validation studies demonstrated a requirement for RUNX2 in the regulation of selected phospho-PR target genes (*SLC37A2*). In vitro mammosphere formation assays support a role for phospho-Ser294-PRs via growth factor (EGF) signaling as well as RUNX2 as potent drivers of breast cancer stem cell fate.

**Conclusions:**

We conclude that PR Ser294 phosphorylation is a common event in breast cancer progression that is required to maintain breast cancer stem cell fate, in part via cooperation with growth factor-initiated signaling pathways and key phospho-PR target genes including *SLC37A2* and *RUNX2*. Clinical measurement of phosphorylated PRs should be considered a useful marker of breast tumor stem cell potential. Alternatively, unique phospho-PR target gene sets may provide useful tools with which to identify patients likely to respond to selective PR modulators that block PR Ser294 phosphorylation as part of rational combination (i.e., with antiestrogens) endocrine therapies designed to durably block breast cancer recurrence.

**Electronic supplementary material:**

The online version of this article (doi:10.1186/s13045-017-0462-7) contains supplementary material, which is available to authorized users.

## Background

Breast cancer is the most commonly diagnosed cancer in women with over 246,000 new cases estimated for 2016 and the second leading cause of cancer-related death in women [1]. Recent publications by The Cancer Genome Atlas (TCGA) Network and others have revealed fundamental molecular characteristics of breast cancer [2–5]. Most notably, the four major breast cancer subtypes (luminal A, luminal B, HER2-enriched, and basal-like) were identified and comprehensively analyzed across datasets that included mRNA expression, protein expression/activation, microRNA expression, DNA copy number variation, DNA methylation, and exome sequencing [5]. These results revealed that breast cancers display a wide range of tumor heterogeneity caused by alterations in multiple factors, including somatically mutated driver genes (e.g., *TP53*, *PIK3CA*, *AKT1*, *CBFB*, *GATA3*, and *MAP3K1*), gene amplifications (*ERBB2*), and hormonally regulated gene programs (driven primarily by estrogen, progesterone, and androgen steroids) [2, 3, 6]. Further genomic analyses reveal additional stratification of breast cancer subtypes. For example, recent TCGA analysis compared ductal and lobular histological subtypes, revealing new molecular factors strongly associated with lobular subtypes, including mutations that lead to activation of AKT and increased FOXA1 expression and activity (i.e., key inputs to amplified estrogen receptor (ER) signaling) [7]. Despite these important findings, targeting the dominant molecular pathways has not been completely successful and many women experience tumor relapse after treatment with targeted therapies (i.e., ~40% of women receiving tamoxifen suffer relapse [8–14]). Thus, a deeper mechanistic understanding of the complex molecular pathways that drive breast cancer progression and how they may emerge accompanied by more precise biomarkers is urgently needed to successfully impede tumor initiation, optimize and customize treatment strategies, as well as prevent disease progression while undergoing long-term (i.e., up to 10 years) endocrine therapy.

**Fig. 1:**
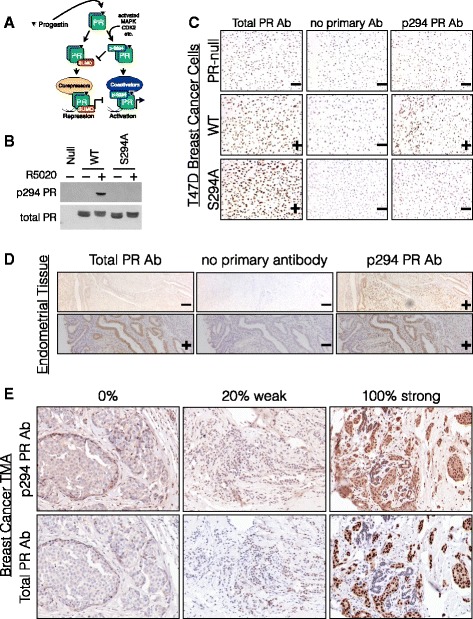
Total PR and phospho-Ser294 PR IHC staining in cell lines, tissue sections, and a breast cancer tissue microarray. **a** Cartoon depiction of PR ligand/kinase-dependent Ser294 phosphorylation, which blocks Lys388 SUMOylation and alters the recruitment of either co-activators or co-repressors, resulting in promoter-selective transcription. **b** PR Ser294 phosphorylation and total PR protein expression levels were measured in T47D breast cancer cell lines by western blotting, with and without progestin R5020 treatment. **c** PR levels were also measured in T47D cells on coverslips using IHC methods. **d** PR Ser294 and total PR protein levels were measured by IHC in a control tissue type (endometrial) to demonstrate effective PR Ser294 (and total PR) antibody specificity and sensitivity. **e** IHC in breast tumor sections (spots) of a tissue microarray. Six representative images demonstrate H-scoring classification: (*column 1*) 0% staining, (*column 2*) 20% positive cells, weak, (*column 3*) 100% positive cells, strong

In breast cancer models, progesterone receptor (PR) target gene selectivity is profoundly affected by cellular context. Like other steroid hormone receptor (SR) family members, PRs are heavily posttranslationally modified and thus act as molecular sensors for abnormally elevated or active signaling pathways. Little overlap exists between PR transcriptomes assayed in normal relative to neoplastic breast cells [15]. In cancer cells, posttranslational modifications (namely, phosphorylation and SUMOylation; see Fig. 1a) create unique PR species whose altered behavior as ligand-dependent transcription factors is predicted to impact tumor initiation and progression [16–20]. As an estrogen-induced target gene and downstream effector of ER action, PR is a master transcription factor that, via both autocrine and paracrine mechanisms, regulates the expansion of luminal and basal mammary epithelial cells during breast development and pregnancy [21–23]. In breast cancer models, numerous ER target genes require PR expression (i.e., via scaffolding actions in ER/PR complexes) for optimal estrogen responsiveness in the absence of exogenously added progesterone [24], while PR can dominantly repress or modify ER actions as part of ER/PR complexes when both ligands (i.e., estrogen and progesterone agonists or antagonists) are combined [25, 26]. These studies illustrate both the complexity and eloquence of SR actions, alone or as frequent collaborators with other SRs and signaling pathways. Recently, PR in cooperation with HER2 signaling was directly implicated as a potent promotor of the “seeds” of early disseminating tumors [27]. These and other studies [28–30] underscore the importance of fully understanding the context(s) for deleterious PR actions relevant to breast cancer biology and particularly to luminal breast tumor progression.

Elevated growth factor signaling is a common early event in breast tumor cells. In this context, multiple signaling pathways dramatically affect both ER and PR actions. For example, PR Ser294 phosphorylation not only is ligand-induced but also occurs via ligand-independent pathways primarily in response to elevated activity of mitogen-activated protein kinases (MAPKs) and/or activated cyclin-dependent kinases (CDKs) in proliferating (i.e., cycling) breast cancer cells. Phosphorylation of PR Ser294 is associated with transcriptional hyperactivity at select phosphorylation-responsive PR target genes required for increased cell proliferation and survival in vitro. Activated phospho-PRs are deSUMOylated and subject to more rapid ligand-dependent receptor downregulation by the ubiquitin proteasome relative to dephosphorylated and SUMOylated PR species [31, 32], explaining the inverse relationship between PR protein levels and peak expression of PR target gene transcripts observed in in vitro models [31, 33–36]. In this heightened signaling context, we predict that a subset of ER+ luminal breast tumor cells that appear to be PR-low (or PR-null) by standard clinical protein immunohistochemistry (IHC) assays may in fact express short-lived phospho-PR species that are SUMO-deficient and thus transcriptionally hyperactive at phospho-PR target genes important for breast cancer cell proliferation and pro-survival.

Herein, we detected Ser294-phosphorylated PRs in 54% of luminal breast cancers (*n* = 209). Notably, phosphorylated PR was significantly associated with invasive lobular carcinoma (ILC), an understudied breast cancer subtype that consists of 10–15% of all ER+ breast cancers. Additionally, consistent with our finding of rapid PR protein loss (i.e., by turnover) of activated (deSUMOylated and phosphorylated) receptors, we detected phospho-PR gene signatures in breast tumors clinically designated as PR-low to PR-null (luminal B) and identified novel gene sets (*HER2*, *PAX2*, *AHR*, *AR*, and *RUNX*) uniquely regulated by modified PRs that are associated with cancer stem cell biology. In vitro mammosphere formation assays confirmed that phosphorylated PRs and RUNX2 are potent drivers of breast cancer stem cell expansion. In sum, our data demonstrate that phospho-Ser294 PRs represent regulatory “gatekeepers” for subsequent expression of key transcription factors implicated in tumor heterogeneity and stem cell biology. Unique phospho-PR target gene sets (including RUNX-regulated genes) may provide useful tools with which to identify patients likely to respond to selective PR antagonists that uniquely block PR Ser294 phosphorylation (i.e., onapristone) as part of novel combination endocrine therapies designed to block ER as well as the gene selective actions of phospho-Ser294 PRs.

## Methods

### Cell culture and reagents

T47D human breast cancer cell lines engineered to stably express PR variants (null, WT, K388R, or S294A) were previously described [32]. T47D cells were maintained in complete minimal essential medium (cMEM) supplemented with 5% fetal bovine serum (FBS), 1% non-essential amino acids (NEAA), 1% penicillin/streptomycin, 6 ng/ml insulin (CellGro, Manassas, VA, USA, catalog #10-010-CV). The T47D cells described above were engineered to also stably express RUNX2 shRNAs via the pLKO.1 knockdown expression vector system, which required 25 μg/ml puromycin for the vector. In various experiments, cells or explants were treated with E2, R5020, mifepristone, aglepristone, or onapristone (kindly provided by Arno Therapeutics, Inc.).

### Breast tumor explants

De-identified breast tumor samples were collected after surgery and immediately processed for tissue explant maintenance on sponges in cell culture medium, as previously described [37]. The samples were derived from six patients pathologically diagnosed with invasive ductal carcinoma and scored positive for ER (94–100%) and PR (1–100%), and negative for HER2 expression. Tissue explants were starved in media containing hormone-stripped FBS for 24 h, and then treated for 48 h with (1) vehicle, (2) 1 nM estradiol, (3) 10 nM estradiol, (4) 1 nM progesterone, and (5) 10 nM progesterone prior to processing for quantitation of Ki-67 levels by IHC. Across treatment conditions, we tested for statistical significance using one-way analysis of variance (ANOVA) followed by pairwise testing of all treatment groups using the TukeyHSD posttest with R statistical software [38]. In additional experiments, six explants were similarly treated with estrogen or progesterone (2 h) but in combination with 1 μM U0126 (to inhibit ERKs 1/2) and phospho-Ser294 PR levels were measured by IHC.

### Immunohistochemistry, immunofluorescence, and immunoblotting

A custom phospho-specific antibody targeting PR Ser294 (clone 8508) was generated in rabbit against peptide sequence: C-PMAPGR(pS)PLATTV-amide (Thermo Fisher Scientific). PR expression was measured by immunohistochemistry methods: 1 × 10^7^ improved MEM (IMEM)-starved T47D cell lines were treated, fixed in 10% neutral buffered formalin for 15 min, embedded in HistoGel (Richard-Allan Scientific), and embedded in paraffin blocks. The samples were sectioned, de-paraffinized, and microwaved for antigen retrieval in 10 mM sodium citrate and stained according to the Vectastain Elite ABC peroxidase (catalog # PK-6101) and ImmPACT DAB (catalog # SK-4105) kits. The slides were counterstained with hematoxylin before imaging.

Immunocytochemistry was performed on T47D cells expressing PR variants to measure total and phospho-Ser294 PR levels. Five hundred thousand cells were grown on coverslips in six-well plates, starved in IMEM plus 5% charcoal-stripped FBS, treated, and fixed with 4% paraformaldehyde for 20 min. The cells were permeabilized with 0.3% Triton X-100 before incubating with total PR (Thermo Scientific, clone Ab8) or custom phospho-Ser294-PR (clone 8508) antibodies. The cells were incubated with fluorescent secondary antibodies (Alexa Fluor 488) and DAPI mounting medium (Life Technologies) before visualizing on a Zeiss microscope with A4 and L5 filter cubes. Immunoblotting was performed as previously described [31].

### Tissue microarray

A breast cancer tissue microarray (TMA) was generated by the University of Minnesota Histology and Immunohistochemistry Laboratory from 209 de-identified breast cancer samples. From this set, 151 tumor samples contained four different pathological regions that were independently included in the array: invasive, inflammatory, DCIS, and adjacent normal-like (normal) tissue within tumor-containing tissue. Patient and tumor characteristics were extracted from pathological reports and used for analysis. Immunohistochemistry was performed on the TMA slides for total PR (antibody clone H190) or phospho-Ser294 PR (antibody clone 8508) expression levels (as described above). The stained slides were scanned using a Huron Technologies TISSUEscope LE by the University of Minnesota Imaging Facility and scored by a pathologist (M.E.S.). The pathologist labeled each tissue spot according to staining percentage (percent of cells positive) and staining intensity (weak, moderate, strong). These two values were combined into a single histology score (H-score) that was used in subsequent analyses [39, 40]. H-scores represent a combination of staining intensity and percent positive cells. H-scores range from 0 to 300, where the staining intensity score (negative (0), weak (1), moderate (2), or strong (3)) is multiplied by the percent positive cells. For example, an H-score of 20 could represent weak staining of 20% of the cells. For multiple regression analysis, H-scores were log2 transformed and standardized prior to model fitting and feature selection. The linear model was fit using the glm function (family = gaussian) in the R statistical software. After backward elimination of non-significant variables, six variables remained significant, resulting in the following regression formula: H-score_PR-Ser294_ = 0.40256 − 0.40579(ER_Pos_) + 0.25459(PR_Pos_) − 0.21988(LN_Pos_) + 0.88609(TumorType_ILC_) − 0.02332(Grade_1_) − 0.18223(Grade_2_) − 0.56517(Tissue_Tumor_). All variables were standardized prior to fitting the model, and the coefficients are plotted with their respective 95% CI.

### Gene expression profiling

For genome-wide microarray expression analysis, T47D cells expressing pIRES-neo3 empty vector, WT PR, or K388R PR were serum starved in modified IMEM (Gibco) for 1 day before treatment. Eight groups were treated with vehicle control, progesterone (10^−8^ M), mifepristone (10^−7^ M), aglepristone (10^−7^ M), onapristone (10^−7^ M), or combined treatment of progesterone + mifepristone, progesterone + aglepristone, or progesterone + onapristone for 6 h before RNA extraction using an RNeasy kit (QIAGEN). DNase I-treated (QIAGEN) RNA samples from triplicate experiments were prepared for expression analysis using the Illumina HT-12v4 beadchip platform according to standard protocols. Raw data from agonist-treated cells (progesterone or R5020) collected from two identically performed independent experiments (from this study and our previous study: GSE34148 [32]) was combined, normalized, and batch corrected to ensure that gene expression values were informative across samples from separate experiments. Data were analyzed within multiple common R [38] and Bioconductor [41] packages. Raw intensities were log2 transformed and quantile normalized using lumi [42] and batch corrected using sva [43], and multiple probes for a single gene were collapsed using genefilter. Differentially expressed genes (pairwise comparisons between all eight groups) were analyzed in limma, where empirical Bayes was used to better estimate the variance of the genes. Biological comparisons (for example, R5020/vehicle) were presented as log2 fold change including the Benjamini and Hochberg (BH)-adjusted *P* value [44] to account for multiple hypothesis testing. Expression data is available in the GEO database (accession: GSE94363).

For reverse transcription quantitative polymerase chain reaction (RT-qPCR) assays, 5 × 105 cells/well were plated in six-well dishes, serum starved in modified IMEM for 1 day before treatment. RNA was extracted using TriPure reagent (Roche), and cDNA was created using the qScript cDNA SuperMix kit (Quanta Biosciences). Relative expression levels were determined by qPCR assays performed on a Roche LightCycler II using SYBR Green Master Mix (Roche). Target gene quantification levels were normalized to the expression of standard housekeeper genes: *TBP*, *ACTB*, *18S*, and/or *GAPDH*.

### Non-negative matrix factorization and hierarchical clustering

Normalized gene expression data (see above) were filtered to isolate only high-variance genes using the Bioconductor package genefilter [45] using an interquartile range cutoff value of 0.85. Non-negative matrix factorization (NMF) [46] was performed within R using the NMF package version 0.20.5 [47], where matrix factors were rank (2–10) and algorithm (brunet or snmf/r) optimized using the nmf function with parameters nrun = 30 and seed = 123456. Based on these results, we chose to fully process our gene expression matrix using the brunet algorithm, rank = 5, nrun = 150, seed = 123456. Hierarchical clustering was performed with normalized expression data (see above), filtered to isolate only high-variance genes (i.e., log2 fold change ≥2 with Benjamini and Hochberg-adjusted *P* value ≤0.01 in any pairwise comparison). Clustering and plots were performed in R (NMF package, a heat map function) using Euclidean distance and UPGMA (average) linkage.

### T47D gene signature analysis within TCGA samples

Gene expression data generated and published by the TCGA consortium [5] was downloaded from the TCGA data portal (https://tcga-data.nci.nih.gov/docs/publications/brca_2012/BRCA.exp.547.med.txt) and quantile normalized using the Bioconductor preprocessCore package [48]. The downloaded data were provided as mean-centered. Tumor sample metadata were downloaded from the TCGA publication including PAM50 molecular subtypes, ER, PR, and HER2 statuses. Tumors classified as luminal A, luminal B, or HER2-enriched and PR-negative (by IHC) were isolated from the dataset and further characterized. For each tumor, we plotted the mean expression value for the collection of genes within a gene set. From these values, the mean and 95% confidence interval was calculated and plotted. Gene sets were derived from experiments in T47D cells, for example, (1) genes upregulated by progestin in T47D cells expressing WT PR versus (2) genes upregulated by progestin in T47D cells expressing KR PR (Additional file 1).

The ductal and lobular TCGA data were downloaded from the Sloan Kettering data freeze (freeze set 3/26/14): http://cbio.mskcc.org/cancergenomics/tcga/brca_tcga [7]. The RNA-seq gene expression values (RSEM) were merged from 705 ductal and lobular samples. Downloaded values were provided as centered z-scores and were log2 transformed across all genes before analysis. The mean expression values for genes within each gene set (PR or random) were plotted for each sample, according to their pathological characteristic (IDC, ILC, or mixed).

### Gene set enrichment analysis

Gene set enrichment analysis (GSEA) software [49, 50] was used to identify gene sets from the Molecular Signatures Database (MSigDB) collections 1–7 that were significantly regulated in cells stably expressing SUMO-deficient PR (K388R) compared to WT PR. Our analysis compared two phenotype groups: KR +R5020/KR −R5020 versus WT +R5020/WT −R5020. GSEA was executed using the default settings, except the permutation type was set to Gene_set with 1000 permutations and the metric for ranking genes was set to Diff_of_Classes because our dataset contained log-scale data.

### Mammosphere culture

#### Primary mammospheres

Adherent cells were washed with PBS and dissociated enzymatically in 0.25% trypsin-EDTA (Invitrogen). The cells were sieved through a 40-μm sieve (BD Falcon) and analyzed microscopically for single cellularity. Single cells were plated in ultra-low attachment plates (Corning) and cultured in a humid incubator. The cells were grown in a serum-free mammary epithelial basal medium (MEBM; Lonza) containing 1% B27 Supplement (Invitrogen), 1% penicillin-streptomycin (Invitrogen), 5 μg/ml insulin (Invitrogen), 20 ng/ml EGF (Sigma), 1 ng/ml hydrocortisone (Sigma), and 100 μM β-mercaptoethanol. Mammospheres were allowed to grow for approximately 14 days. Mammosphere forming efficiency (MFE, %) is calculated by the number of mammospheres per well/number of cells seeded per well.

#### Secondary mammospheres

Primary mammospheres were collected by centrifugation (5 min, 1000 rpm) and dissociated enzymatically in 0.25% trypsin-EDTA. Cells were sieved through a 40-μm tip strainer (Bel-Art SP Scienceware) and analyzed microscopically for single cellularity. Single cells were plated in ultra-low attachment plates and cultured in a humid incubator. The cells were grown in conditioned media for approximately 14 days. The conditioned media consists of a 1:1 mixture of mammosphere media (described above) and media from cultured parental cells. MFE (%) is calculated by the number of mammospheres per well/number of cells seeded per well.

## Results

### A majority of breast tumors contain phospho-Ser294 PR

We previously demonstrated functional roles for phosphorylation of PRs by mitogenic protein kinase pathways commonly elevated in breast cancers, including MAPKs, CDKs, and CK2 [33, 51–55]. These events are predicted to enable gene promoter selection by uniquely modified PR species according to cell context (Fig. 1a). To demonstrate the prevalence of PR Ser294 phosphorylation in human luminal breast tumors in vivo, we completed IHC staining of a tissue microarray (TMA) containing 209 patient breast tumors (split into 2754 tissue spots) for both total PR and phospho-Ser294 PR (Table 1). Note that phospho-Ser294 antibodies are unable to distinguish between PR isoforms. However, PR-B but not PR-A is primarily phosphorylated on Ser294 in breast cancer models [33, 56]. We thus validated total and phospho-Ser294-specific PR antibodies by performing western blotting and IHC on PR-null and PR+ T47D cells containing either WT PR-B or S294A mutant PR-B (Fig. 1b) and further optimized PR staining protocols for IHC using human PR+ healthy uterine tissues (Fig. 1c). For the majority of tumor samples in our breast cancer TMA, four pathological regions of each tumor were identified by a clinical pathologist (designated as invasive, inflammatory, DCIS, or normal-like) and represented as separate tissue spots. Following staining with either total PR or phospho-Ser294 PR antibodies, a histology score (H-score) was calculated for each tissue spot based on the percent of positively stained cells and their staining intensity (strong, moderate, or weak, Fig. 1d, e) [39, 40].

**Table 1:** Breast cancer tissue microarray patient characteristics

	Number (*n* = 209)	Percent
ER/PR status		
ER-positive	163	78.0
ER-negative	40	19.1
PR-positive	120	57.4
PR-negative	83	39.7
ER-positive and PR-positive	117	56.0
ER-positive and PR-negative	46	22.0
ER-negative and PR-negative	37	17.7
Unknown	6	2.9
HER2 status		
HER2-positive	59	28.2
HER2-negative	140	67.0
Intermediate	1	0.5
Unknown	9	4.3
Lymph node status		
LN-positive	75	35.9
LN-negative	111	53.1
Unknown	23	11.0
Grade		
1	34	16.3
2	96	45.9
3	66	31.6
Unknown	13	6.2
Tumor type		
Invasive ductal carcinoma	168	80.4
Invasive lobular carcinoma	21	10.0
DCIS	2	1.0
Other	14	6.7
Unknown	4	1.9
Tumor volume		
<10 cm^3^	64	30.6
≥10, <20 cm^3^	48	23.0
>20 cm^3^	68	32.5
Unknown	29	13.9

The number and percentage of patient breast tumors included in the TMA study, stratified by various common breast tumor features

**Fig. 2:**
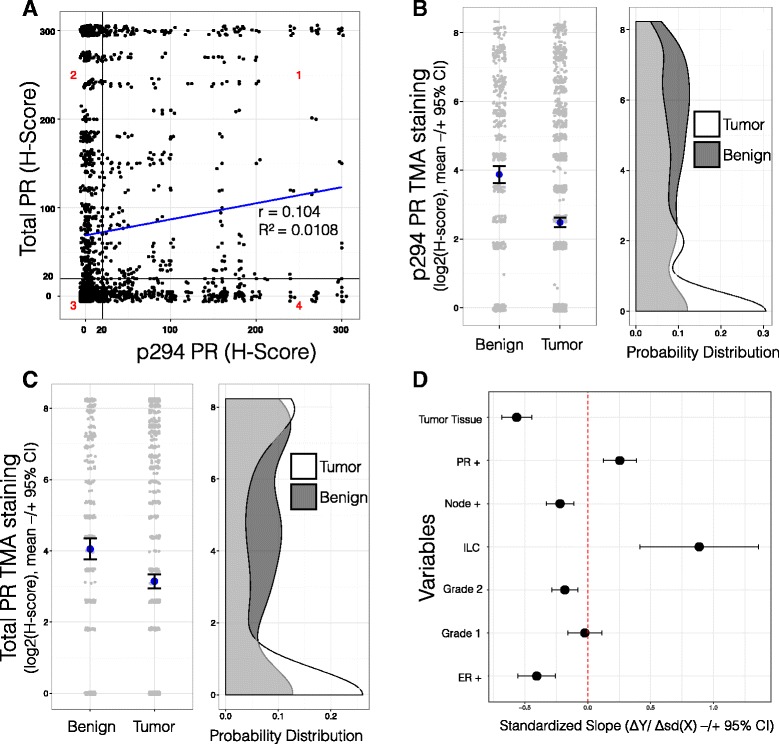
PR Ser294 phosphorylation and total PR H-scores are not correlated, and PR Ser294 phosphorylation H-scores were negatively associated with various tumor characteristics. **a** H-scores for total PR expression and phospho-Ser294 PR were compared among individual tumors spots from our TMA study. A Pearson correlation was calculated (*r* = 0.104, *R* ^2^ = 0.0108). Tissue spots considered “positive” had an H-score of >20. Four quadrants were labeled (1−4) and discussed in the text. **b** *Left*: TMA spots were separated based on benign breast tissue (BBT) or tumor tissue (TT) pathological classification, and PR Ser294 phosphorylation H-scores were plotted (*gray dots*) with mean values (*blue dots*, ±95% CI). *Right*: H-score densities reveal wide distributions for both groups, but H-scores among TT samples are skewed toward zero. **c** Same analysis as Fig. 2b, except for total PR H-scores. **d** With IHC staining scores and patient metadata from our breast cancer TMA study, we used multiple regression to predict PR Ser294 H-scores from various factors. Significant variables (*P* < 0.05) have 95% CIs non-overlapping with the zero line

As determined by our pathologists (see “Methods”), H-scores ranged from the minimum to maximum (0–300) and samples with H-scores ≥20 were classified as positive. Overall, ~70% of tumors in this representative luminal tumor TMA stained positive for total PR. Of these PR+ samples, 54% were also positive for phospho-Ser294 PR expression. The percentage of tumors completely negative (H-score = 0) for total PR staining was 15%, and that for phospho-Ser294 PR staining was 8%. Notably, total PR expression was not substantially correlated with the presence of Ser294-phosphorylated PR (*r* = 0.104) in individual tumor spots, with some tumors having completely opposite total and phospho-Ser294 PR H-scores (Fig. 2a). Thus, our results reveal that phospho-Ser294 PRs can be readily detected in a significant subset of individual tumors that appear to express relatively low levels of total PR (quadrant 4, 19%). Conversely, ~23% of tumors (quadrant 2) expressed high total PR and low phospho-Ser294 PR. Thirty-nine percent of tumors were negative for both total PR and phospho-Ser294 PR (quadrant 3) while 17% of tumors were positive for both total and Ser294-phosphorylated receptors (quadrant 1). Positive staining for phospho-Ser294 PR was greatest in tissue spots pathologically classified as “Normal” (54%; normal-like tissue within tumor-containing tissue), followed by “DCIS” (47%), “Inflammatory” (43%), and “Invasive” (38%) sections, having the lowest H-score levels for expression of phospho-Ser294 PR. Positive staining for total PR levels by tissue type were as follows: Normal (72%), DCIS (55%), Inflammatory (53%), and Invasive (52%). These data indicate that total PR and phospho-Ser294 PR staining are not directly related in this TMA as measured using distinct antisera and that PR levels are diminished in tissues with invasive characteristics relative to normal tissues or regions of DCIS.

We predicted that lowered PR expression in tumors relative to benign breast tissue (BBT) is indicative of heightened (i.e., activated) PR transcriptional activity that occurs during the process of tumor progression. We thus compared the levels of phospho-Ser294 PR or total PR expression between these two tissue classifications. IHC scoring was completed by an independent breast cancer pathologist, who also classified the tissue spot as BBT or tumor tissue (TT). As predicted, H-scores among the BBT samples were significantly greater than those among the TT samples (Ser294: *P* < 2.2e−16, Mann-Whitney test, Fig. 2b; total PR: *P* < 7.8e−06, Mann-Whitney test, Fig. 2c). These data suggest that while both total and phospho-PR expression is varied within regions of established tumors, greater levels of both total and phospho-PR are typically found in epithelial layers displaying early lesions or normal-like pathology. Overall, the majority of tumors (TT) represented in this TMA contained less total PR/phospho-Ser294 PR relative to BBT. Interestingly, at least 21 tumor samples of invasive lobular carcinoma (ILC) were represented in our TMA as 468 spots. These tumors also expressed heterogeneous levels of total and phospho-Ser294 PRs. Phosphorylated PR levels were similar within each quadrant: 1–4 (29, 20, 21, and 27%), again bearing no direct relationship to total PR levels (Additional file 2 and further discussed below).

We next probed the relationship between PR Ser294 phosphorylation and the available patient tumor characteristics (Table 1). We investigated whether any of the tumor characteristics (independent variables) could predict PR Ser294 phosphorylation H-scores (dependent variable) using a multiple regression method. All independent variables were initially included in the model, and non-significant variables were removed stepwise by backward elimination until a core set of significant variables remained. In this model, in addition to PR positivity (at clinical diagnosis), only invasive lobular carcinoma (ILC tumor type) was a significant indicator of PR Ser294 phosphorylation. Multiple factors were negative predictors of PR Ser294 phosphorylation, including tumor tissue pathology (vs. benign breast tissue), lymph node positivity (vs. node negativity), grade 3 status (vs. grade 2 or 1), and ER positive status (vs. negative status at clinical diagnosis) (Fig. 2d). These findings suggest that PR Ser294 phosphorylation is a relatively common but early event in breast cancer development. The presence of phospho-PR species may indicate that these early lesions contain sufficient levels of local progesterone and/or express activated MAPK or CDK signaling (i.e., downstream of growth factor receptors, for example) relative to tissues that are strongly PR+ but lack appreciable levels of phospho-Ser294 PR (i.e., expressing largely inactive/dephosphorylated and stable receptors) [19, 31].

### Progesterone treatment of breast tumor explants cultured ex vivo drives proliferation and induces PR Ser294 phosphorylation

**Fig. 3:**
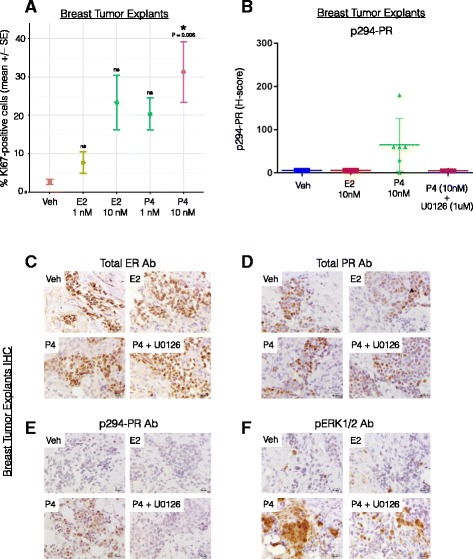
Proliferation and biomarker expression in breast tumor explants in response to estrogen (E2), progesterone (P4), or combined P4 + U0126 treatment. **a** Post surgery, breast tumors were dissected and prepared for tissue explant experiments. Tumors were cut into small fragments and placed on sponges soaked in tissue culture medium. Sections were treated with vehicle (ethanol), E2 (1 or 10 nM) or P4 (1 or 10 nM) for 48 h. Tissue sections were then fixed, embedded, and processed for Ki-67 IHC staining. The percent of Ki-67-positive cells were plotted (mean ± SE). Comparing the groups via one-way ANOVA, followed by TukeyHSD posttest, indicated that only the P4 (10 nM) treatment was significantly different from vehicle (*P* = 0.0061, *n* = 6 explants per treatment condition). **b** Breast tumor explants were treated with vehicle, estradiol (*E2*, 10 nM), progesterone (*P4*, 10 nM), or a combination of P4 and MAPK inhibitor U0126 (1 μM) for 2 h. Explants were fixed, paraffin embedded, and stained for phospho-Ser294 PR expression, and H-scores were plotted. **c**–**f** Representative tumor explant IHC images after staining for total ER, total PR, pSer-294 PR, and phospho-ERK1/2 expression

Because PR expression is primarily estrogen-induced in a majority of PR+ tissues and cancer models, isolating the unique contributions of progesterone/PR in breast cancer biology can be difficult to study in breast cancer models without the confounding (i.e., proliferative) effects of estrogen/ER. We thus tested the proliferative response to progesterone treatment in ex vivo 3D cultures of human breast tumor tissue (i.e., tumor explants). Fresh tumor fragments from ER+/PR+ tumors were dissected into 1-mm^3^ sections and maintained on gelatin sponges submerged in cell culture medium as previously described [37, 57, 58]. Explants were treated with 1 or 10 nM estrogen or progesterone for 48 h before the tumor fragments were embedded in paraffin, sectioned, and analyzed by IHC for Ki-67 expression. ER+ tumor explants treated with progesterone (10 nM) but not estrogen (1 and 10 nM) had a significantly higher percentage of Ki67-positive cells (a marker of cell proliferation), compared to vehicle treatment (*P* = 0.006, ANOVA with TukeyHSD posttest; *n* = 6) (Fig. 3a). Note that 10 nM estrogen alone induced a weak proliferative response in breast tumor tissue explants [25] that was similar to the proliferative trend observed herein (Fig. 3a). These data show that progesterone (P4) treatment alone significantly stimulates proliferation in ex vivo breast tumor tissue samples.

We previously demonstrated a proliferative and pro-survival role for MAPK-dependent phosphorylation of PR on Ser294 in breast cancer cells [32]. To assess whether PR Ser294 is a regulated phosphorylation site in human tumors ex vivo, we again employed the human tumor explant model as above (Fig. 3a). ER+ luminal tumors were maintained as explants as above and instead treated with either vehicle or progesterone (10 nM) for 2 h in the presence or absence of the MEK1/2 inhibitor U0126 (1 μM) prior to IHC staining using specific antibodies for total and phospho-Ser294 PR as well as ER-alpha and phospho-ERK1/2 (*n* = 6; see “Methods”). As predicted, progesterone treatment induced robust PR Ser294 phosphorylation that was blocked by inclusion of the MEK inhibitor U0126 (Fig. 3b). IHC staining demonstrated that all explants were ER+ and PR+ (representative examples are shown; Fig. 3c, d). However, only progesterone treatment (P4) induced robust phosphorylation of PR Ser294 that was accompanied by activation of ERK1/2 (representative examples are shown; Fig. 3e, f). Phospho-ERK1/2 staining confirmed that progesterone (but not estrogen) activated ERKs 1/2 and that U0126 also blocked the majority of hormone-induced ERK 1/2 activity in breast tumor tissues (Fig. 3f). These data demonstrate that progesterone, at a physiologic dose, is a potent mediator of breast tumor cell proliferation (independent of estrogen) in a model system that maintains breast tumor 3D structure, microenvironment, and epithelial cell polarity (known factors required for PR expression and paracrine actions [59, 60]) and indicate that PR-dependent transcriptional programs (i.e., that drive proliferation) including those enacted by MAPK-dependent phosphorylation of PR on Ser294 are likely to be activated in human breast cancers cultured ex vivo.

### Mifepristone and aglepristone, but not onapristone, induce PR Ser294 phosphorylation and act as partial agonists

**Fig. 4:**
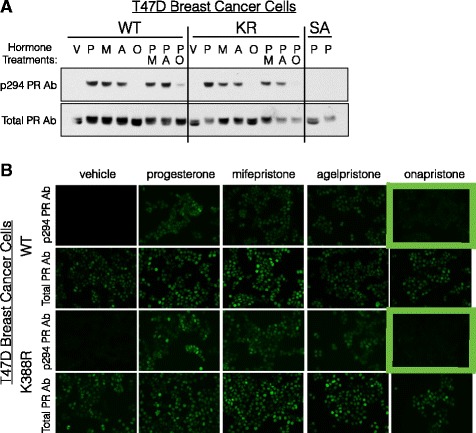
Select PR antiprogestins, mifepristone and aglepristone, induce PR Ser294 phosphorylation, but onapristone does not. **a** T47D cells expressing wild type (*WT*) PR or Ser294/SUMO-deficient PR (*KR*) were treated with vehicle (*V*), progesterone (*P*), mifepristone (*M*), aglepristone (*A*), onapristone (*O*), or a combination of progesterone and each antiprogestin. Cells were harvested for western blotting analysis and revealed that both mifepristone and aglepristone induce PR Ser294 phosphorylation, whereas onapristone does not. Co-treatment of progesterone and mifepristone or aglepristone also induce Ser294 phosphorylation, whereas onapristone blocks Ser294 phosphorylation even in the presence of progesterone. **b** Similar to western blotting analysis, T47D cells were treated as described above and analyzed for PR expression by immunofluorescence. Again, only onapristone effectively blocked PR Ser294 phosphorylation in both cells expressing WT or KR PR (highlighted by *green box*)

PR antagonists have been examined for the treatment of PR-positive breast cancer with results comparable to that of tamoxifen [61, 62]. These agents have not been prioritized primarily because first-generation antiprogestins exhibited cross-reactivity with both glucocorticoid and androgen receptors accompanied by intolerable toxicities in early trials. In addition, extensive luminal breast cancer heterogeneity may limit the ability to observe a subset of PR-driven breast cancers without patient selection (for review, see [19]). In this case, PR target gene expression may provide an accurate means of predicting which breast tumors are likely to be influenced by PR-driven biological pathways enacted by active phospho-Ser294 PRs. To probe changes in PR target gene expression in the presence or absence of commonly used PR ligands (R5020, RU486), including diverse antiprogestins (aglepristone, onapristone) currently in development for clinical use, we utilized the well-characterized model system of T47D breast cancer cells, stably expressing either unmodified wild-type (WT) PR-B or a transcriptionally hyperactive form of deSUMOylated K388R PR-B (KR; this receptor faithfully mimics phosphorylated PR-B with regard to target gene selection) [19, 31]. We initially tested whether the antiprogestins mifepristone (also called RU486), aglepristone, and onapristone alter PR Ser294 phosphorylation in these T47D breast cancer models. Cells were treated for 1 h with vehicle, progesterone, mifepristone, aglepristone, or onapristone, and whole cell lysates were processed for western blotting or immunofluorescence (IF) analysis (Fig. 4a, b). Consistent with previous reports, progesterone treatment stimulated PR Ser294 phosphorylation in cells expressing either unmodified PR (WT) or SUMO-mutant (KR) PR. Similarly, in cells expressing either WT or activated KR PR, mifepristone and aglepristone stimulated robust PR Ser294 phosphorylation, whereas onapristone alone had no effect on PR Ser294 phosphorylation. Liganded PRs exhibited a slight gel mobility upshift (Fig. 4a) due to multiple phosphorylation events known to occur within the PR N-terminus. Greater loss of total KR PR protein was also apparent in the presence of progesterone and selected antiprogestins relative to liganded WT PR, consistent with increased turnover of deSUMOylated active receptors relative to intact WT PRs [31]. Notably, only onapristone blocked Ser294 phosphorylation in the presence of progesterone. These data show that PR Ser294 phosphorylation is stimulated by multiple ligands including the common PR antagonists mifepristone and aglepristone. However, onapristone treatment does not permit Ser294 phosphorylation, even in the presence of progesterone, predicting that cells treated with this ligand will exhibit distinct gene expression profiles relative to other ligands (i.e., both agonists and antagonists) that stimulate PR Ser294 phosphorylation.

**Fig. 5:**
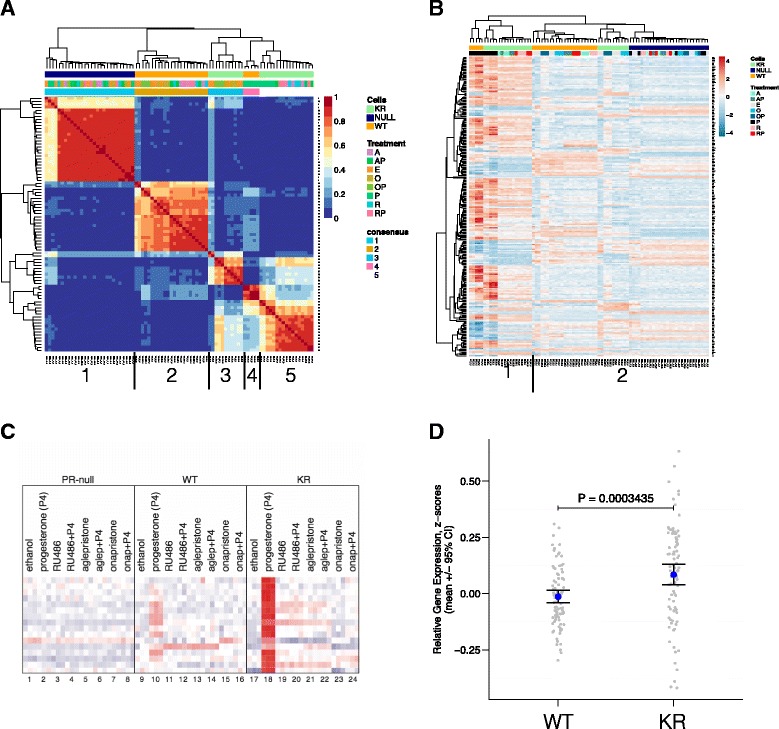
Gene expression analysis in T47D cells treated with various ligand combinations demonstrates unique promoter selection. **a** Gene expression arrays were used to measure global changes in gene expression levels in T47D cells treated with different PR ligands: vehicle, progestin (P), mifepristone (M), aglepristone (A), onapristone (O), P + M, P + A, or P + O. Genes under high variance across these samples were isolated, and expression values were used for NMF clustering. Presented is the consensus matrix that indicates five major clusters are present in the samples. **b** Using the gene expression dataset (log2 fold change ≥2, BH *P* value ≤0.01), multiple sample comparisons (i.e., vehicle vs. P) were made and genes that were significantly regulated were isolated (*rows*). These genes were clustered via unsupervised hierarchal clustering methods, and two major branches were identified (clusters 1 and 2). In addition, sub-branches can also be seen, suggesting a total of five independent sample groups. **c** We identified 16 PR target genes that were specifically regulated in T47D breast cancer cells expressing Ser294 phosphorylation/SUMO-deficient PR (KR) and not regulated by WT PR (that is not phosphorylated and SUMOylated). In addition, we identified 101 genes that were specifically upregulated by WT PR (non-phosphorylated and SUMOylated PR) (not shown). **d** We compared the average expression of these 16 genes or 101 genes in the published TCGA breast cancer cohort of PR-negative tumors. Despite all of these tumors being PR-negative (by clinical IHC diagnosis), the activated PR target genes (KR) are expressed at significantly higher levels compared to genes upregulated by WT PR (*P* = 0.0003435)

PR ligand-mediated promoter selectivity remains understudied, especially in the context of antiprogestins and posttranslationally modified PR species. Breast tumors clearly express phosphorylated PR molecules (Fig. 1e and 2a) predicted to be deSUMOylated and transcriptionally hyperactive at a subset of SUMO-sensitive and phosphorylation-dependent gene promoters [32]. To further explore altered phospho-PR promoter selectivity (Fig. 1a), we conducted global gene expression analyses in T47D breast cancer cells expressing either WT or K388R PR-B receptors treated as above (Fig. 4a). PR-null cells or cells expressing unmodified WT or SUMO-deficient KR PR species were serum starved (24 h) prior to ligand treatment (6 h). The cells were then treated with vehicle control (ethanol), progesterone (P), mifepristone (M), aglepristone (A), onapristone (O), or combined (progesterone agonist plus each antagonist) treatments of P + M, P + A, or P + O. Total RNA was collected and subjected to microarray gene expression analysis using the Illumina HT-12v4 beadchip platform. Our normalized gene expression dataset included 84 different samples under the above treatment conditions, necessitating identification of commonly regulated sample clusters (i.e., groups of similarly regulated samples) in an unbiased manner. First, we isolated genes under high variance and performed NMF analysis (a clustering method that does not depend on prior knowledge of the sample labels or molecular classifications of breast cancer; see “Methods”) [46, 63–65]. The resulting consensus matrix indicated five uniquely regulated sample clusters within the gene expression dataset (Fig. 5a). Annotating the consensus matrix with cell line and treatment labels revealed five sample clusters: (1) PR-null cells (all treatments), (2) WT cells (all antiprogestin and vehicle treatments), (3) KR cells (onapristone and vehicle treatments), (4) WT cells (progestin treatments), and (5) KR cells (progestin, aglepristone, and mifepristone treatments) (Fig. 5a). These data clearly show that in cells expressing activated PR (i.e., KR or deSUMOylated PR), the antiprogestins mifepristone and aglepristone significantly regulated a similar gene expression program to that of progestin agonists (progesterone or R5020). Further, depending on the phosphorylation/SUMOylation status of PR, the receptor regulates completely different target genes when bound to different classes of antiprogestin (mifepristone/aglepristone vs. onapristone). The antiprogestins aglepristone and mifepristone substantially regulate multiple genes in cells expressing KR PR, but not WT PR, whereas in contrast, onapristone does not substantially regulate PR target genes in either cell line. These data suggest that the status of PR posttranslational modifications can substantially impact PR target gene selectivity in a ligand-selective manner. Namely, deSUMOylated (KR) PRs recognize selected antagonists (mifepristone and aglepristone) as potent receptor agonists relative to onapristone.

In addition to unsupervised NMF clustering (above), we performed differential gene expression analysis between various biologically interesting cell line/treatment comparisons and identified 251 genes that were up- or downregulated greater than twofold in any comparison (Fig. 5b). Using this set of PR regulated genes, unsupervised hierarchal clustering revealed that progestin-treated samples clustered closely together in cells expressing either WT or KR. Hierarchical clustering revealed two major branches of closely related samples: (1) WT cells treated with progestin and KR cells treated with progestin, mifepristone, or aglepristone and (2) WT cells treated with any antiprogestin or vehicle, KR cells treated with onapristone or vehicle, and all PR-null cells regardless of treatment (Fig. 5b). This result demonstrates that various antiprogestins have different transcriptional effects depending on the dominant PR species. However, based on this clustering analysis, all samples (expressing WT or KR PR) treated with onapristone were closely related (and members of the second branch), suggesting that onapristone effectively inhibited PR (either WT or KR) transcriptional activity comparable to the level found in PR-null (control) cells (Fig. 5b, cluster 2, right). Furthermore, importantly, onapristone did not stimulate SUMO-deficient PR target gene expression in KR-containing T47D cells, as did mifepristone and aglepristone (Fig. 5b, cluster 1).

### PR-low (by IHC) breast tumors significantly express “activated PR” target gene signatures

We demonstrated that PR transcriptional activity is directly linked to rapid proteasome-mediated turnover of ligand-bound receptors [33, 51] and that ligand-dependent PR downregulation is greatly augmented by phosphorylation of PR Ser294 in response to activated MAPK or CDK2 signaling pathways [33]. To address this context-dependent complexity, we identified activated PR target genes that were specifically regulated in cells expressing SUMO-deficient PRs (as markers of phosphorylated or hyperactivated PR transcriptional activity) and examined their average expression levels in the TCGA breast cancer patient cohort [5]. First, we isolated only luminal A, luminal B, and HER2-enriched tumors that were diagnosed as ER+ but PR-negative by clinical IHC, as we suspected some of these tumors would contain undetected but hyperactivated PRs. Next, in these tumors, we compared the expression of genes known to be primarily upregulated by deSUMOylated (i.e., phosphorylated) activated PRs relative to genes known to be regulated by SUMOylated PRs (Fig. 5c) [32]. As we predicted, tumors clinically classified as PR-negative were characterized by elevated expression of activated PR target genes (Fig. 5d). These exciting results suggest that a cohort of “PR-negative” breast tumors assigned using standard clinical IHC protocols in fact express significantly high levels of phospho-Ser294 PR target gene mRNA transcripts whose collective expression (i.e., the activated PR transcriptome) signifies the presence of activated phospho-Ser294 PRs. Our data suggest that modified PRs cannot be reliably detected in the clinical setting by measurement of PR protein expression (as determined by IHC) as the sole marker of PR activity. Indeed, in our TMA results (above), PR Ser294 phosphorylation and total PR expression were not substantially correlated in individual tumors.

### Phospho-Ser294 PR target genes are more highly expressed in ILC tumors compared to IDC tumors

**Fig. 6:**
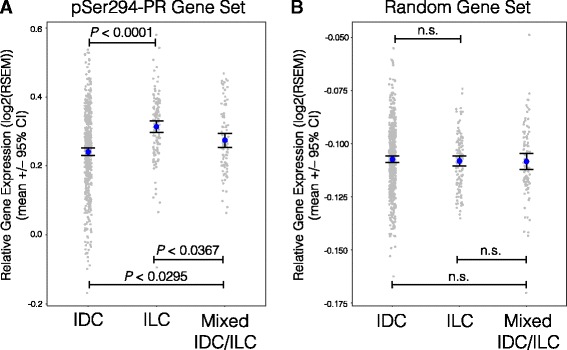
The phospho-Ser294 PR gene set is upregulated in ILC breast tumors. **a** Mean gene expression values for a phospho-Ser294 PR gene set (see Fig. 5c) were plotted (*gray dots*) for tumors classified as IDC, ILC, or mixed IDC/ILC by the TCGA project. The mean of all values within each tumor subset were plotted (*blue dots*, ±95% CI), and groups were statistically compared using ANOVA with TukeyHSD posttest. Adjusted *P* values are displayed. **b** A control analysis was repeated with a random set of 150 genes

Our TMA analysis (Fig. 2d) revealed that phospho-Ser294 PR expression was significantly associated with invasive lobular carcinoma (ILC), when compared to other tumor types included in the model. TCGA recently published a comprehensive analysis that directly compared ILC and IDC breast tumors [7]. We utilized this large independent tumor cohort to further probe the relationship between phospho-Ser294 PR signaling in lobular and that in ductal tumors. We compared the expression levels of a phospho-PR gene set in ILC, IDC, and mixed IDC/ILC tumors from the TCGA dataset (Fig. 6a). The phospho-PR gene set (upregulated by phospho-PR/SUMO-deficient PR, Fig. 2c) was significantly more expressed in the ILC tumors, when compared to the IDC and mixed ILC/IDC tumors (*P* < 0.0001, ANOVA with TukeyHSD posttest; *n* = 705). These data suggest that genes regulated by phospho-Ser294 PR may drive cells toward the ILC tumor lineage, as compared to ductal subtypes.

### GSEA reveals mechanisms for SUMO-deficient PR transcriptional activation

Whole genome expression analysis allows the identification of functional characteristics within a dataset that will lead to new hypotheses about the model system. Notably, complex cellular responses often result from subtle changes in gene expression levels of multiple genes acting in concert to mediate an important biological outcome. Thus, we employed gene set enrichment analysis (GSEA) to identify gene sets significantly enriched by progesterone or in selective progesterone receptor modulator (SPRM)-treated groups relative to controls and specifically regulated by phospho-Ser294 PRs. All seven gene set collections from the Molecular Signatures Database (MSigDB, version 4) [49] were analyzed independently among pairwise sample/treatment comparisons. Comparisons of data derived from cells expressing SUMO-deficient/phospho-Ser294 mimic (KR) PR to unmodified WT-PR (−/+SPRMs) revealed numerous significant (nominal *P* < 0.05, FDR < 0.25) gene sets. In addition to predicted PR target gene sets (Additional file 3A), we again observed ERBB2/HER2 as a phospho-Ser294-PR-driven gene set (Additional file 3B) as previously described [32]. In addition, PAX2 and aryl hydrocarbon receptor (AHR) as well as androgen receptor (AR) and glucocorticoid receptor (GR) gene sets (which share similar consensus sequences to that of PR) [66] were significantly upregulated in cells expressing KR-PR (+SPRMs) but not in similarly treated cells expressing WT-PR (Additional file 3C–E), suggesting that genes regulated by active SUMO-deficient or phospho-Ser294 PRs are more likely to contain classical steroid receptor binding motifs near the transcriptional start site and may thus have DNA binding priority relative to unmodified WT PRs (i.e., primarily de-phosphorylated but capable of undergoing ligand-induced SUMOylation). Finally, we observed six significantly enriched EVI-1/RUNX (also called AML) gene sets uniquely regulated in cells expressing KR-PR (+SPRMs) relative to cells expressing WT-PR (+SPRMs) (Additional file 3F), suggesting that phosphorylated PRs and RUNX factors may cooperate on selected target genes. Three RUNX transcription factors have been described and are important mediators in multiple cancers, including AML. Notably, RUNX transcription factors are primarily expressed in stem cells and regulate stem cell renewal [67].

#### Functional cooperation between phospho-Ser294 PR and RUNX2

**Fig. 7:**
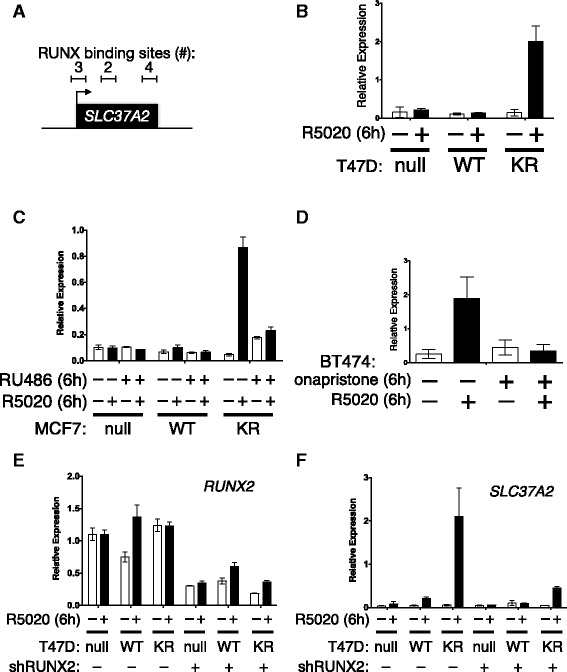
RUNX2 may facilitate SUMO-deficient PR target gene expression. **a** *SLC37A2* genomic region contains multiple RUNX binding motifs and other regulatory regions (CpG islands and other transcription factor binding hot spots). The number (*#*) of RUNX binding motifs within three major regions are listed. **b** *SLC37A2* expression in T47D cells was measured after treatment with progestin (R5020) by RT-qPCR. **c** *SLC37A2* expression in MCF-7 cells was measured after treatment with progestin (R5020) and/or antiprogestin (mifepristone) by RT-qPCR. **d** *SLC37A2* expression in BT474 cells was measured after treatment with progestin (R5020) and/or antiprogestin (onapristone) by RT-qPCR. **e** T47D cells expressing WT or KR PR were engineered to stably express shRNAs targeting RUNX2, resulting in approximately 50% reduction of RUNX2 mRNA levels. **f** In cells stably expressing shRNAs targeting RUNX2, expression of the KR PR target gene *SLC37A2* was significantly reduced

The above GSEA results suggest that SUMO-deficient phospho-Ser294 PRs regulate a set of genes also regulated by RUNX factors. PR cooperation with one or more RUNX factors may be a mechanism for promoter selection by uniquely modified receptors. The family of RUNX transcription factors (RUNX1, 2, and 3) has complex roles in development and tumor formation with both tumor-suppressive and tumor-promoting activities. Interestingly, phenotypes associated with RUNX2 expression in mammary epithelial cells closely resemble phenotypes dependent on PR as well as progestin-mediated gene expression (namely, cyclin D1 expression, proliferation, luminal progenitor cell maintenance, and alveolar expansion during mammary gland development; see “Discussion”). From our GSEA results, we identified *SLC37A2*, a candidate PR target gene containing multiple RUNX2 binding motifs immediately upstream and within the gene (Fig. 7a). *SLC37A2* is a glucose-6-phosphate transporter expressed in monocytes as well as breast and cervical tissues. Although no studies have been conducted in cancer models, *SLC37A2* is associated with at least 17 other public datasets that define stem cell genes or proteins [68]. We thus measured PR/progestin-dependent regulation of *SLC37A2* mRNA expression in multiple cell line models. In T47D cell models, *SLC37A2* expression was robustly stimulated by progestin in cells expressing SUMO-deficient PR, but not in cells expressing WT PR (Fig. 7b). Similarly, in MCF-7 cell line models overexpressing SUMO-deficient K388R PR, *SLC37A2* expression was upregulated by progestin exposure but blocked by mifepristone (Fig. 7c). Interestingly however, as predicted from our gene array studies, mifepristone exhibited weak partial agonist activity in cells expressing KR PRs (compare RU486 treatments across cell lines). We previously showed that BT474 breast cancer cells (luminal B; ER+/PR+/ERBB2+) superinduce selected SUMO-sensitive (activated) PR target genes upon progestin treatment relative to other PR+ cell line models, presumably because kinase pathways downstream of HER2 (i.e., MAPKs) input to persistent PR Ser294 phosphorylation [32]. In unmodified BT474 cells, progestin exposure resulted in highly phosphorylated PR that turned over rapidly, characteristic of PRs with heightened transcriptional activity [51]. In these cells, progestin treatment also stimulated robust *SLC37A2* mRNA expression that was effectively blocked by treatment with onapristone (Fig. 7d). We then tested the requirement for RUNX2 expression in transcriptional responses to progestin by knocking down RUNX2 in T47D cells using shRNAs. Although T47D cells remained relatively resistant to RUNX2 loss in our hands, RUNX2 expression was reproducibly reduced by approximately 50% upon expression of specific shRNAs relative to shRNA controls (Fig. 7e). Knockdown of RUNX2 greatly attenuated induction of *SLC37A2* expression in cells expressing KR-PR and treated with progestin relative to controls (Fig. 7d). These data demonstrate that PR cooperation with RUNX2 contributes to *SLC37A2* expression as part of a unique phospho-Ser294 PR transcriptome in breast cancer cells and illustrate the impact of context-dependent cell signaling on PR actions.

#### PR Ser294 phosphorylation is required for formation of secondary mammospheres

**Fig. 8:**
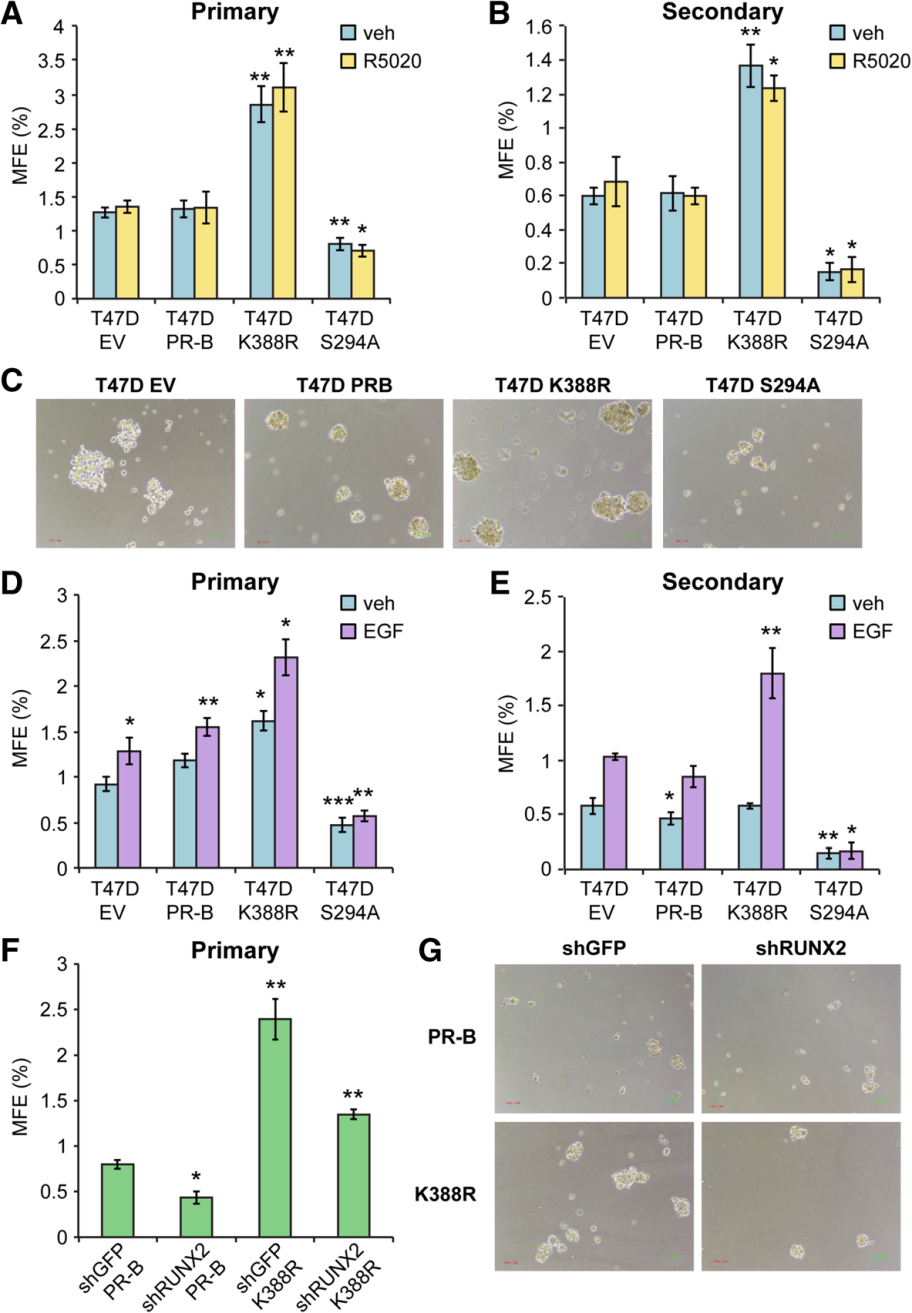
Mammosphere formation in T47D cells (empty vector, PR-B, PR-B K388R, and PR-B S294A). **a** Primary and **b** secondary mammospheres in T47D cells overexpressing empty vector, PR-B, PR-B K388R, or PR-B S294A and plotted as a percentage of mammosphere forming efficiency (*MFE*; see “Methods”). Cells were treated with vehicle (EtOH) or R5020 (10 nM). **c** Images of primary mammospheres (vehicle) from **a**. **d** Primary and **e** secondary mammospheres in T47D cells treated with vehicle (water) or EGF (20 ng/ml). Mammospheres were allowed to grow for 14 days prior to counting. **f** Primary mammospheres in T47D cells (PR-B or K388R) expressing shGFP or shRUNX2. **g** Images of primary mammospheres from **f**. Data is represented as the average ± SD of three readings. *p < 0.05, **p < 0.01, ***p < 0.001 compared to empty vector control (vehicle)

**Fig. 9:**
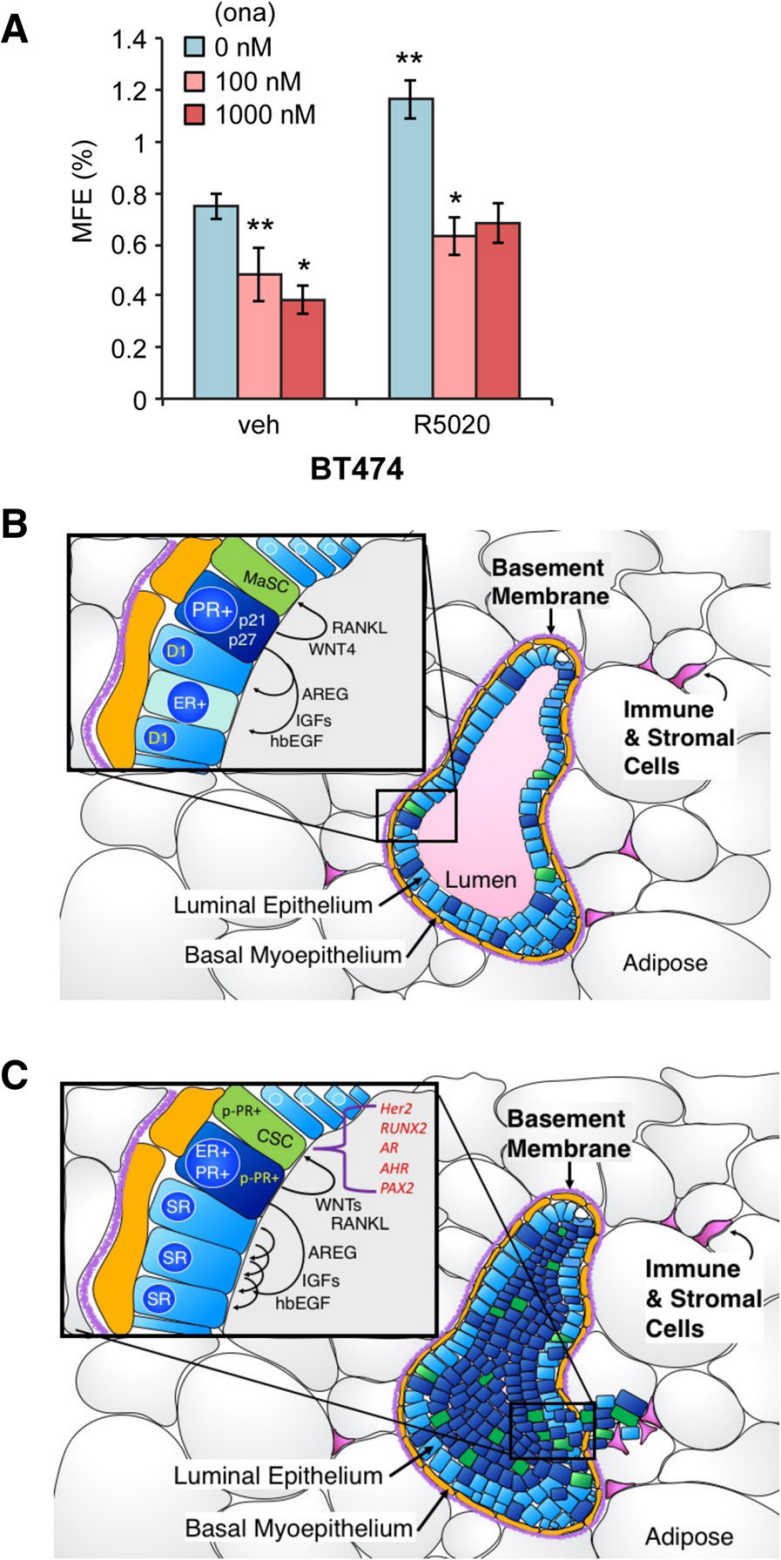
Primary mammosphere formation in unmodified HER2+ BT474 breast cancer cells. **a** Primary mammospheres in HER2+ BT474 cells expressing endogenous estrogen receptor (ER) and progesterone receptor isoforms (PR-A and PR-B) plotted as a percentage of mammosphere forming efficiency (*MFE*; see “Methods”). Cells were treated with vehicle (EtOH) control or R5020 (10 nM) without or with increasing concentrations of the type II antiprogestin onapristone (0, 100, or 1000 nM). Data is represented as the average ± SD of three readings. *p < 0.05, **p < 0.01, ***p < 0.001 compared to vehicle control. Secondary mammospheres failed to form in onapristone-containing media (not shown). **b**, **c** Model depicting PR action in normal breast (**b**) vs. during neoplastic luminal tumor progression (**c**). Phosphorylation of PR Ser294 and p-PR target gene expression (HER2, RUNX2, AR, AHR, PAX2) in the cancer stem cell (CSC) or neighboring tumor cell compartments may occur during early luminal tumor progression of ER+/PR+ (luminal A type) breast cancers that progress toward ER+/PR-low (and HER2+) (luminal B type) tumors that are CSC-rich and thus more likely to become endocrine-resistant. Early addition of anti-progestins to anti-estrogen/ER-based therapies may prevent or delay the onset of endocrine therapy-resistant luminal breast cancer recurrence

Interestingly, HER2, PAX2, AHR, AR, and RUNX factors have each been implicated in cancer stem cell biology ([27, 67, 69–71] and see “Discussion”). Further, these factors may cooperate; AR/RUNX2 complexes are important drivers of prostate cancer stem cell expansion [72]. Mammosphere assays provide an assay of stem cell potential, wherein formation of secondary mammospheres (i.e., derived from dissociated and serially passaged primary mammospheres) is a definitive assay of the ability of minority breast cancer cell stem cells within a heterogeneous population to expand and reestablish as E-cadherin-positive spheres able to grow in suspension culture following long-term serial passage as non-adherent cells [73]. To demonstrate a role for phosphorylated PRs in breast cancer stem cell biology, we performed mammosphere assays using our well-defined T47D cell model systems expressing either empty vector (EV PR-null), unmodified WT PR-B, point mutant KR PR-B (K388R), or point mutant S294A PR-B missing the consensus MAPK phosphorylation site Ser residue (Fig. 8). Equal numbers of T47D cells were inoculated into primary mammosphere assays (i.e., suspended cell culture) in defined media, and mammosphere numbers were scored after 2 weeks by manual counting using a uniformly scaled grid; primary mammospheres were gently dissociated and reseeded in order to form secondary mammospheres (see “Methods”). Interestingly, cells expressing either EV or WT PR produced similar basal numbers of primary (~25) and secondary (~10) mammospheres, while cells expressing KR (phospho-mimic) PR consistently produced 55–70 primary mammospheres and 35–50 secondary mammospheres (Fig. 8a, b). Surprisingly, both primary and secondary mammosphere formation was greatly attenuated in cells expressing S294A PR relative to controls and cells expressing KR PR. Interestingly, PR-null (vector control) cells formed small “flat” or “non-spheroid” clumps of loosely associated cells with raged or rough edges relative to cells expressing WT PR-B, which formed small round and smooth mammospheres (Fig. 8c). Notably, cells expressing KR PR formed larger mammospheres relative to cells expressing WT PR, in sharp contrast to cells expressing S294A PR, which formed few very small mammospheres (Fig. 8c). Addition of either estrogen (1 nM; not shown) or progesterone (10 nM; shown) to mammosphere culture media had no significant effect on total numbers in any condition. However, removal (and add-back) of EGF to the mammosphere culture media demonstrated a clear requirement for growth factor signaling (Fig. 8d, e). These data suggest that formation of secondary mammospheres, a definitive assay of stem cell outgrowth, is largely dependent on the presence of signaling inputs (EGF) to phospho-Ser294 PRs in T47D breast cancer cells but does not require exogenously added progesterone. Further, the finding that expression of S294A PR attenuated mammosphere formation to levels below that of either PR-null or WT PR-containing cells in EGF-containing media suggests a dominant negative effect of this mutant receptor, perhaps via interaction with other steroid receptors such as ER or AR (see “Discussion”). We next tested the ability of PR-B+ T47D cells stably expressing either control shRNA (shGFP) or RUNX2 shRNA to form mammospheres (Fig. 8f, g). Again, cells expressing K388R PR-B formed larger and significantly greater numbers of primary mammospheres relative to cells expressing unmodified (WT) PR-B. Knockdown of RUNX2 greatly attenuated the formation of primary mammospheres in T47D cells expressing either WT PR-B or K388R PR-B, rendering the assay of secondary mammospheres infeasible. Finally, in a similar set of experiments, we validated our results in unmodified ER+/PR+ BT474 cells. These cells express high levels of activated HER2 and thus more closely resemble luminal B-type breast cancers, but express endogenous ER and both PR isoforms (PR-A and PR-B). In this “high kinase” context, PRs are readily phosphorylated on Ser294 (ref Knutson BCR 2012). Notably, BT474 cells exhibited a relatively high level of basal primary mammosphere formation that was further elevated in the presence of progestin (Fig. 9a). Treatment with onapristone (an antiprogestin that blocks PR Ser294 phosphorylation) effectively reduced both basal and progestin-stimulated primary mammosphere formation. As with RUNX2 knockdown studies, secondary mammospheres failed to form in the presence of onapristone (not shown).

Collectively, our data suggest that phospho-PRs are key gatekeepers that enable breast tumor progression via induction of multiple signaling pathways, including those required for outgrowth of breast cancer stem or progenitor cells (Fig. 9b, c). Identification of phosphorylated receptors in human tumors and discovery of phospho-PR-regulated pathways (i.e., including HER2, RUNX2, AR, AHR, and PAX2) suggest novel ways to specifically target breast cancer stem cell (CSC) outgrowth as part of durable breast cancer therapies. We conclude that antiprogestins that block PR Ser294 phosphorylation should be included “up-front” as part of routine endocrine therapies for women undergoing long-term management of ER+ luminal breast cancer.

## Discussion

Our data provide insight into how progestin treatment may block proliferation in some strongly ER+/PR+ breast cancers (containing PRs capable of undergoing regulated SUMOylation, a modification that is primarily transcriptionally repressive at SR target genes and required to repress ER-alpha and other SR-dependent transcriptional events), while stimulating proliferation in others (containing modest levels of phosphorylated and SUMO-deficient PRs that are active drivers of unique cancer transcriptomes). Additionally, our findings support a growing body of evidence implicating PR as a master regulator of cell fate of both normal mammary epithelial and cancer stem/progenitor cell populations and reveal a key role for Ser294-phosphorylated PRs in this aspect of PR-driven cell biology. Ultimately, the transcriptional activity and biological actions of PRs are profoundly influenced by context. Herein, we identified a subset of PR target genes that can be used as biomarkers reflective of activated PR expression (i.e., independently of clinically derived PR status as defined by IHC-based methods). Using breast cancer mRNA expression data from the TCGA project, we determined that activated PR target genes were significantly upregulated in ILC as well as clinically determined PR-negative luminal patient samples (compared to gene sets specifically regulated by inactive or stabilized and abundant receptors). These data suggest that a subset of breast cancer patients whose tumors are clinically classified as PR-negative may have cancers driven in part by modest levels of highly transcriptionally active PRs that go undetected by clinical standards. Alternatively, abundant phospho-PRs may reside in minority cancer cell populations or “PR+ islands” within largely PR-null tumors (Figs. 1 and 2) capable of early dissemination [27]. Patients harboring such tumors are strong candidates for antiprogestin therapy, including onapristone or similar agents that block PR Ser294 phosphorylation.

As an ER target gene product, PR is classically used as a biomarker of functional ER and thus indicative of a high likelihood of response to ER-targeted endocrine therapies [74, 75]. Tumors defined as ER+ PR+ HER2– are usually less aggressive and classified within the luminal A or B subtypes. Of these, ER+/PR-low or ER+/PR-null tumors (i.e., luminal B subtype) are more likely to become endocrine-resistant (Fig. 9c). The presence of PR can profoundly modify ER behavior and cellular responses to estrogen, in part by direct ER/PR interactions [25, 76]. Modest levels of PR-B, but not progesterone, were required for estrogen-induced changes in global gene expression associated with breast tumor progression to endocrine resistance and poor disease outcome [24]. In contrast, estrogenic responses were inhibited when ER+/PR+ breast cancer cells and breast tumor explants were exposed to both hormones; however, relatively high hormone concentrations were used to demonstrate these effects [25, 26]. Like estrogen (alone), progesterone (alone) is a potent driver of breast cancer cell proliferation (Fig. 3a). PR+ but ER-null mammary gland progenitor cells exist, suggesting unique roles for PR that are independent of ER; PR+ bipotent progenitor cells are estrogen-insensitive, while estrogen regulates PR expression only in mature luminal cells [77]. Progesterone but not estrogen has emerged as a key mediator of both normal and neoplastic mammary gland stem cell expansion [78–81]. Our studies strongly implicate Ser294 phosphorylated PRs in this activity (Fig. 8). The finding that PR Ser294 phosphorylation is widely observed in breast tumors and is primarily found in premalignant regions suggests that this modification of PR is a relatively early event in tumor progression that is likely most relevant to luminal A to luminal B transition, wherein PR expression appears diminished as PR target gene expression peaks. Notably, expression of the PR target gene, RANKL, also primarily occurs in early-stage premalignant epithelial layers (i.e., DCIS, and normal-like regions) [78, 82]. Recently, Hosseini et al. [27] showed that mRNA levels of *pgr* and progesterone-induced paracrine signals (PIPS; *rankl* and *wnt4*) were upregulated in cells from early mammary lesions of BALB-NeuT mice. These PIPS were required for early lesion cell migration as well as sphere formation.

We found that commonly used PR ligands (agonists and antagonists alike) induce PR Ser294 phosphorylation and phospho-PR target gene expression (Figs. 4 and 5). Indeed, the partial agonist activity of antiprogestins appears to map to SUMO-deficient/phosphorylated receptors. Only onapristone was effective in blocking Ser294 phosphorylation and gene expression in cells expressing either wild type (WT) PR or SUMO-deficient (KR) PR (Figs. 4 and 5). In breast cancer cells expressing KR PR, mifepristone and aglepristone stimulated considerable Ser294 phosphorylation and gene regulation, suggesting these antagonists may be less effective in cells that contain the highly transcriptionally active deSUMOylated PR. Antiprogestin therapies are being actively studied for breast (and other) cancers (reviewed in [19, 83]); therefore, a more comprehensive understanding of the differences in transcriptional regulation by these ligands (in relation to PR posttranslational modifications) will be critical. Our data (Fig. 5) demonstrate that different antiprogestins have unique gene regulatory action depending on the status of PR posttranslational modifications. Both mifepristone and progestin agonists (progesterone or R5020) upregulated similar genes in cells expressing SUMO-deficient (phospho-mimic) PRs, suggesting that mifepristone is a poor antiprogestin in that context. However, mifepristone treatment of cells containing WT PR (capable of SUMOylation) was far less likely to stimulate the expression of progestin-regulated PR target genes, making it a useful antagonist in that context. These results suggest that successful therapies for breast cancer patients using antiprogestins should consider the status of PR posttranslational events. Our data may explain why mifepristone (RU486) has not been successful in clinical trials for breast cancer, considering that our TMA revealed that PRs in a majority of breast tumors are phosphorylated on Ser294, a posttranslational event predicted to confer partial agonist activity to ligands of this class. As such, alternative antiprogestin therapies (i.e., onapristone or SPRMs that block PR Ser294 phosphorylation) may be more successful to silence the transcriptional action of activated phospho-PRs.

We previously defined phospho-Ser294 PRs as major transcriptional drivers of gene programs significantly associated with HER2/ERBB2 signaling in breast cancers [32]. Related to our findings, PR and HER2 were shown to cooperate as required mediators of early breast cancer dissemination and metastasis [27]. Herein, our GSEA analyses confirmed that phospho-PRs significantly induce expression of HER2-associated gene sets and demonstrated that phospho-PR target genes also include key mediators of cancer stem cell biology, including PAX, AHR, AR, and RUNX family members (Additional file 3; Fig. 7). SUMO-deficient or phospho-Ser294 PR target genes may be co-regulated by one or more of these transcription factor families. Notably, PAX2 is overexpressed in >50% of breast cancers and was required for progesterone-stimulated lateral side-branching and lobular development in a murine Pax2-knockout model [84]. Pax2 knockout in murine mammary glands phenotypically resembled PR or Wnt4-knockout mice [21, 85]. These data indicate that PR-driven pathways important during mammary gland development may remain active during breast tumor progression. Aryl hydrocarbon receptor (AHR) is a transcription factor member of the nuclear receptor (NR) superfamily expressed in female reproductive tissues that interacts with multiple environmental toxins as ligands, resulting in AHR translocation to the nucleus, where it dimerizes with AHR nuclear translocator (ARNT). This triggers upregulated expression of cytochrome P450 enzymes that help metabolize a variety of compounds. Environmental toxins can modulate reproductive functions and alter homeostasis of many endocrine functions in the reproductive tract largely because ligand-activated AHR can interfere with SR signaling. Thus, AHR is known to disrupt ER and AR target gene expression [86]. For example, ligand-bound AHR/ARNT binds specific motifs positioned near ER binding motifs in the promoters of multiple ER target genes, disrupting ER-mediated transcription [87, 88]. Similarly, our GSEA results demonstrate that activated PRs drive expression of important SR-regulated target genes that can also be disrupted by AHR/ARNT complexes (Additional file 3). Thus, phosphorylated and SUMO-deficient PR may interact preferentially with AHR/ARNT-repressed genes. Notably, like PR, which clearly contributes to maintenance of the adult mammary stem cell compartment ([80] and for review, see [89]), AHR signaling has recently been implicated in regulation of stem-like cell expansion in breast cancer models [90]. Similarly, AR is highly expressed (80%) across all breast cancer subtypes and also negatively and positively interacts with ER signaling [91–94].

We observed six significantly enriched ecotropic viral integration site 1 (EVI1) or RUNX (also called AML) gene sets regulated in cells expressing KR-PR + progestin, compared to cells expressing WT-PR + progestin (Additional file 3). EVI1 is a transcription factor that is primarily expressed in stem cells and regulates stem cell renewal (for review, see [67]). The oncoprotein is well studied in acute myeloid leukemia (AML) and, when highly expressed, confers poor outcome. AML is primarily caused by gene translocations between strong promoters and the *EVI1* and runt-related transcription factor 1 (*RUNX1*) genes. Three RUNX transcription factors have been described and are important mediators in multiple cancers in addition to AML. In particular, our GSEA results demonstrate that SUMO-deficient phospho-PR regulates a set of genes that contain RUNX DNA binding motifs in their promoters, suggesting that SUMO-deficient or phosphorylated PR and RUNX factors may cooperate in a way that WT PR (i.e., SUMOylated) does not. Both RUNX1 and RUNX3 have tumor suppressor roles in breast cancers, while RUNX2 is tumor-promoting (for review, see [95]). Namely, RUNX2 is an osteoblast differentiation transcription factor expressed in developing breast epithelial cells that is enriched in the mammary stem cell population responsible for terminal end bud differentiation [96, 97]. As a proof of principle, we validated a requirement for RUNX2 in phospho-Ser294 PR target gene regulation (i.e., using the *SLC37A2* gene identified in our arrays; Fig. 7) as well as in primary mammosphere formation (Fig. 8). Previous reports have shown that RUNX2 interacts with estrogen receptor (ER), androgen receptor (AR), and the glucocorticoid receptor (GR) to facilitate steroid hormone-mediated transcriptional activity. Curiously, we were unable to co-IP RUNX2 and PR from breast cancer cell whole cell lysates or detect them as co-associated factors at PRE sites by ChIP assays, suggesting that these factors function in the same pathway but may interact indirectly or may associate transiently or successively via binding to separate or distant sites in chromatin (a topic for further study). In mice, RUNX2 was required for proper alveolar differentiation [98], a property shared by PR-B [99]. *Runx2* gene deletion resulted in disrupted alveolar progenitor cell populations, differentiated cell type histology, reduced levels of cell proliferation, reduced tumor burden, and longer overall survival in a mouse model of breast cancer [98]. In addition, deletion of *RUNX2* in human breast cancer cells resulted in reduced tumorigenic properties, such as invasion and migration. Conversely, aberrant overexpression of *RUNX2* induced EMT-like changes in normal mammary glands and also caused cells to remain in a less-differentiated state with elevated expression of cyclin D1, a well-known PR-B (and other SRs) target gene and transcriptional co-regulator of phospho-PR at cancer-relevant PR-B/cyclin D1 target genes [98, 100]. Our data show that RUNX2 is essential for mammosphere formation in PR-B+ cells (Fig. 8); we were unable to obtain sufficient cell numbers from primary mammospheres of RUNX2 knockdown models for assay of secondary mammospheres. Interestingly, while mammosphere formation was insensitive to added hormones, EGF was required for spheroid formation in breast cancer cells expressing K388R (phospho-mimic) PRs (Fig. 8d, e). These data suggest that cells growing in suspension no longer require exogenously added hormones but instead rely on growth factors to cue context-dependent (i.e., MAPK-dependent) phospho-PR actions, including gene expression of RUNX2. EGF-induced steroid hormone biosynthesis is a topic of further study as a potential mechanism of SR action in breast cancer spheroids carried in media lacking exogenously added hormones (Fig. 8). A deeper understanding of RUNX2 as a mediator of PR actions in breast cancer also warrants future study.

## Conclusions

In sum, PR is emerging as a major mechanistic player that mediates early breast tumor progression in part via “feeding” the stem cell compartment (i.e., via paracrine signals); our data support a requirement for phosphorylation of PR Ser294 in this activity as an important gatekeeper of breast cancer cell fate and expanded tumor heterogeneity. Most notably, in addition to strongly ER+/PR+ lobular breast cancers, we observe expression of KR-specific target genes in human breast tumors clinically determined to be PR-negative. We propose that this PR signature is an important biological “marker” of activated phospho-PR species that undergo rapid protein loss due to turnover, an event that may precede loss of PR mRNA expression in more advanced and strongly HER2+ tumors [31–33]. Previous clinical trials using antiprogestins demonstrated poor response rates in PR+ tumors. These agents may have stimulated PR phosphorylation and unwanted target gene expression. Additionally, these early trials primarily targeted PR in strongly ER+/PR+ (luminal A) tumors. While this was a logical approach based on high expression of PR protein as a biomarker, our studies suggest a far more complex scenario in which luminal B (PR low) patients are the correct cohort for antiprogestins. The recent finding that PR and HER2, a primary pathway induced by phospho-Ser294 PR [32], were requisite mediators of early breast cancer dissemination and metastasis [27] underscores the relevance of our work. Clearly, a paradigm shift to activated PR as measured by the presence of phospho-PR species or phospho-PR target gene sets (in addition to HER2) is urgently needed.

## Additional files

Additional file 1: Gene sets derived from T47D cells expressing WT PR and KR PR. These gene sets were used in the analysis described in Fig. 5c, d. (A) 16 genes were discovered to be highly upregulated by progestin in cells expressing KR PR, compared to WT PR. These genes were also not regulated by other PR ligands. (B) 101 genes were discovered to be highly upregulated by progestin in cells expressing WT PR, compared to KR PR. These genes were also not regulated by other PR ligands. (XLSX 28 kb)

Additional file 2: PR Ser294 phosphorylation and total PR H-scores in only invasive lobular carcinoma (ILC) TMA tumor spots. H-scores for total PR expression and phospho-Ser294 PR were compared among individual tumors spots from our TMA study. A Pearson correlation was calculated (*r* = 0.041, *R* ^2^ = 0.0017). Tissue spots considered “positive” had an H-score of >20. Four quadrants were labeled (1–4) and discussed in the text. (PDF 28 kb)

Additional file 3: Gene set enrichment analysis (GSEA) in T47D breast cancer cells comparing KR + progestin vs. WT + progestin treatment groups. GSEA identified significantly regulated gene sets in the KR + progestin samples when compared to the WT + progestin samples. Five gene sets from the c3 MSigDB collection and one from the c6 collection are shown: (A) genes containing PR DNA binding motifs, (B) genes upregulated after ERBB2 overexpression in MCF-7 cells, (C) genes containing androgen receptor DNA binding motifs, (D) genes containing PAX family DNA binding motifs, (E) genes containing AHR/ARNT DNA binding motifs, and (F) genes containing AML1/RUNX binding motifs. These upregulated gene sets contain (respective) DNA binding motifs (above) near their transcriptional start sites, suggesting that these factors are important co-transcriptional regulators with PR in T47D cells expressing Ser294/SUMO-deficient PR (KR), compared to WT PR. (PDF 257 kb)

## Abbreviations

ANOVA: Analysis of variance
AR: Androgen receptor
ER: Estrogen receptor
GR: Glucocorticoid receptor
GSEA: Gene set enrichment analysis
HER2: Human epidermal growth factor receptor 2
IDC: Invasive ductal carcinoma
IHC: Immunohistochemistry
ILC: Invasive lobular carcinoma
MAPK: Mitogen-activated protein kinase
NMF: Non-negative matrix factorization
PR: Progesterone receptor
SUMO: Small ubiquitin-like modifier
TCGA: The Cancer Genome Atlas
