# Supplementary figures and images for: Posttranslationally modified progesterone receptors direct ligand-specific expression of breast cancer stem cell-associated gene programs
Source: J Hematol Oncol. 2017 Apr 17;10:89. doi: 10.1186/s13045-017-0462-7 (PMC5392969; doi:10.1186/s13045-017-0462-7)

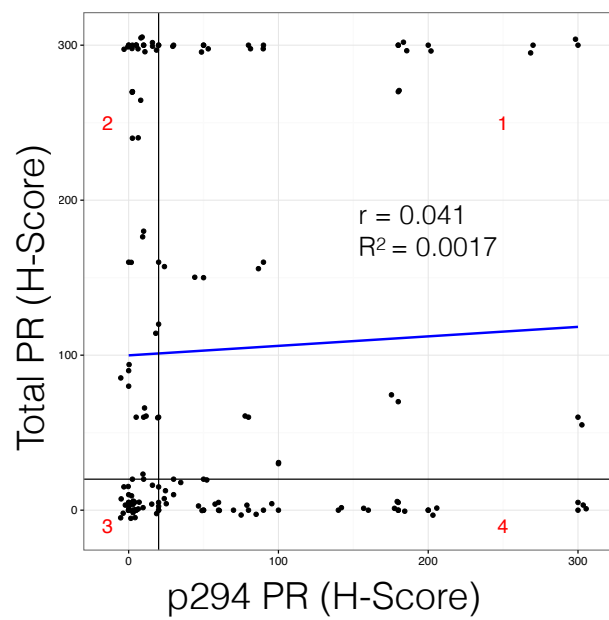

Supplement: Supplementary file 2 — PR Ser294 phosphorylation and total PR H-scores in only invasive lobular carcinoma (ILC) TMA tumor spots. H-scores for total PR expression and phospho-Ser294 PR were compared among individual tumors spots from our TMA study. A Pearson correlation was calculated (r = 0.041, R 2 = 0.0017). Tissue spots considered “positive” had an H-score of >20. Four quadrants were labeled (1–4) and discussed in the text. (PDF 28 kb) [file 13045_2017_462_MOESM2_ESM.pdf]

**A**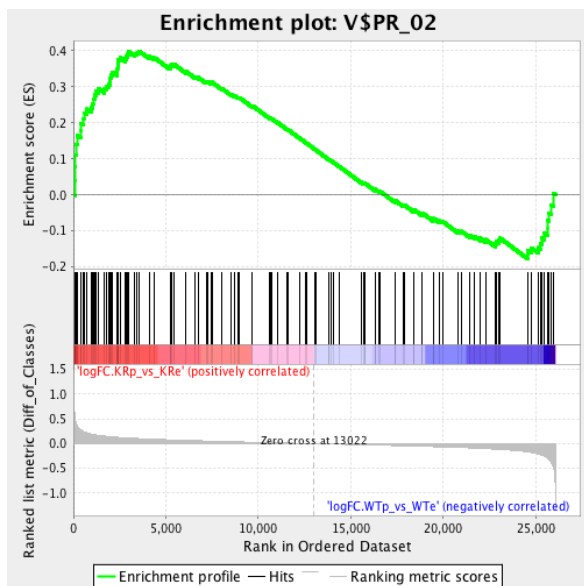**B**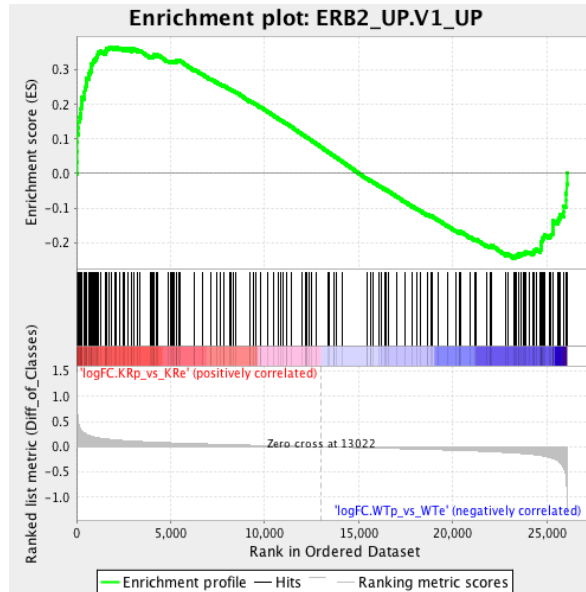**C**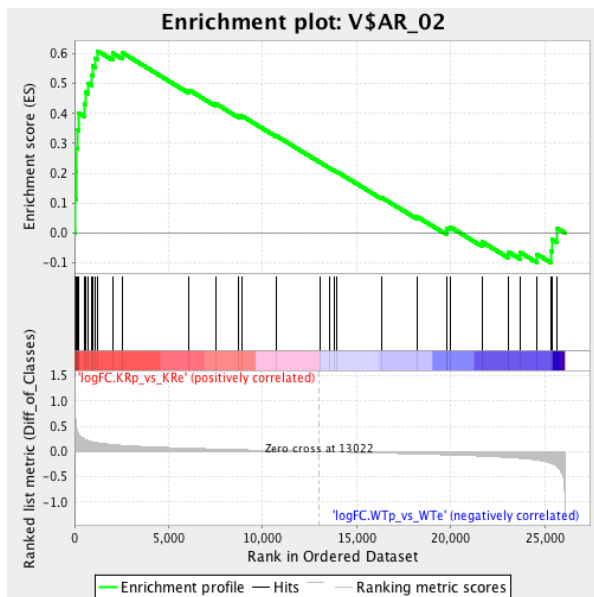**D**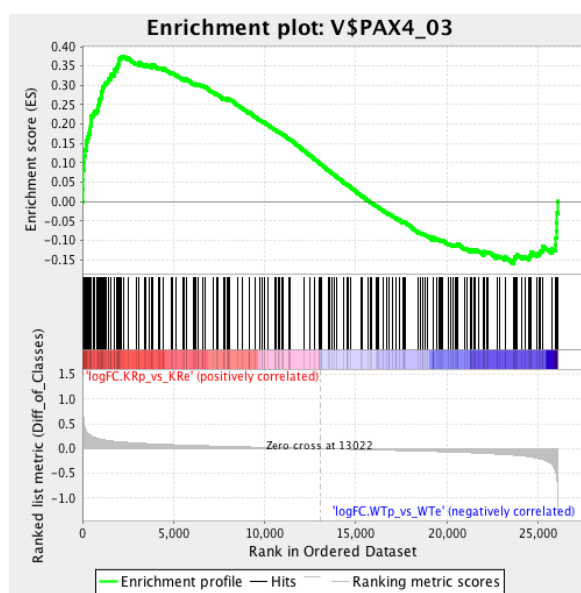**E**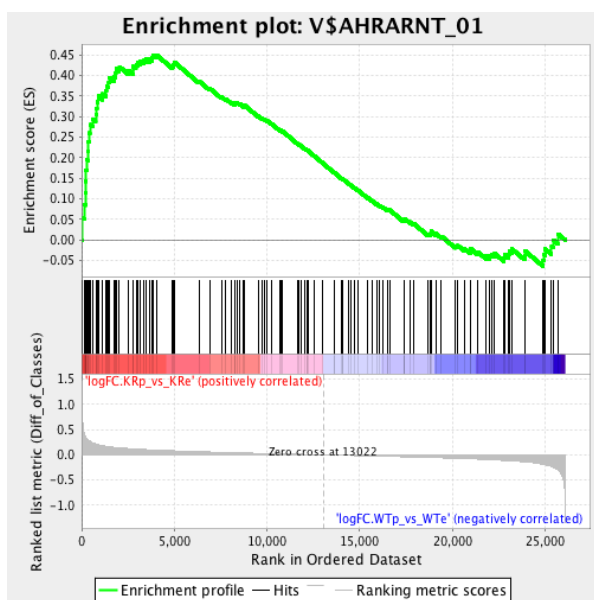**F**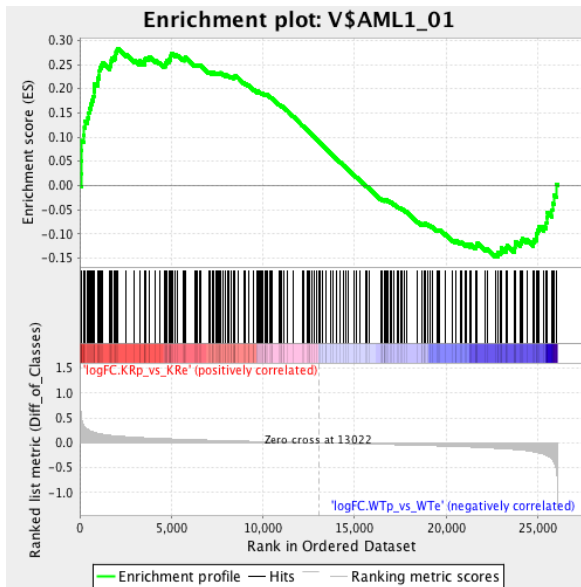

Supplement: Supplementary file 3 — Gene set enrichment analysis (GSEA) in T47D breast cancer cells comparing KR + progestin vs. WT + progestin treatment groups. GSEA identified significantly regulated gene sets in the KR + progestin samples when compared to the WT + progestin samples. Five gene sets from the c3 MSigDB collection and one from the c6 collection are shown: (A) genes containing PR DNA binding motifs, (B) genes upregulated after ERBB2 overexpression in MCF-7 cells, (C) genes containing androgen receptor DNA binding motifs, (D) genes containing PAX family DNA binding motifs, (E) genes containing AHR/ARNT DNA binding motifs, and (F) genes containing AML1/RUNX binding motifs. These upregulated gene sets contain (respective) DNA binding motifs (above) near their transcriptional start sites, suggesting that these factors are important co-transcriptional regulators with PR in T47D cells expressing Ser294/SUMO-deficient PR (KR), compared to WT PR. (PDF 257 kb) [file 13045_2017_462_MOESM3_ESM.pdf]
